# Ectopic invasive ACTH-secreting pituitary adenoma mimicking chordoma: a case report and literature review

**DOI:** 10.1186/s12883-023-03124-7

**Published:** 2023-02-23

**Authors:** Yan Li, Jian-Guo Zhu, Qing-Quan Li, Xiao-Jing Zhu, Ji-Hong Tian

**Affiliations:** 1 Department of Radiology, the Second Affiliated Hospital of Nanjing Medical University, No. 121, Jiangjiayuan Road, 210011 Nan Jing, P.R. China; 2 Department of Neurosurgery, the Second Affiliated Hospital of Nanjing Medical University, Nan Jing, P.R. China; 3 Department of Pathology, the Second Affiliated Hospital of Nanjing Medical University, Nan Jing, P.R. China; 4 Department of Radiotherapy, the Second Affiliated Hospital of Nanjing Medical University, Nan Jing, P.R. China

**Keywords:** Ectopic pituitary tumor, Invasive, Sphenoid sinus, Clivus, Case report

## Abstract

**Background:**

Ectopic pituitary adenoma (EPA) is defined as a special type of pituitary adenoma that originates outside of the sellar region, is extra- or intra-cranially located, and without connection to normal pituitary tissue. EPA is extremely rare, with most cases presented as case reports or small case series. Due to nonspecific symptoms and laboratory indicators, the preoperative diagnosis, treatment and management for EPA remain challenging.

**Case presentation:**

Here, we report the imaging phenotype and pathological findings of a case of invasive EPA in a 47-year-old woman. A preoperative non-contrast CT scan revealed a 5.8 × 3.6 × 3.7 cm soft tissue mass located in the sphenoid sinus and clivus. MRI showed an ill-defined solid mass with heterogeneous signals on T1-weighted and T2-weighted images. The mass displayed infiltrative growth pattern, destroying bone of the skull base, invading adjacent muscles and encasing vessels. The patient underwent partial tumor resection via transsphenoidal endoscopic surgery. Pathological examination led to diagnosis of ectopic ACTH-secreting pituitary adenoma. Post-surgery, the patient received external beam radiotherapy.

**Conclusion:**

EPA with invasive growth pattern has rarely been reported. The imaging phenotype displays its relationship to the pituitary tissue and surrounding structures. Immunohistochemical examination acts as a crucial role in differentiating EPA from other skull base tumors. This case report adds to the literature on EPA by summarizing its characteristics alongside a review of the literature.

## Background

Pituitary adenoma originates from anterior pituitary cells and is the most common tumor in the sellar region, accounting for about 10–15% of all intracranial tumors [1, 2]. Most pituitary adenomas located in the sellar turcica, and those occurring outside the sellar without direct connection to the normal pituitary tissues, are called ectopic pituitary adenomas (EPA). EPA is an extremely rare tumor with about 100 cases reported in the English literature [3–6]. The majority of EPA cases are benign, but a few cases can be malignant or undergo a malignant transformation. In this report, we present a case of EPA of large size and obvious invasiveness, alongside a review of the literature on this rare tumor.

## Case presentation

**Fig. 1:**
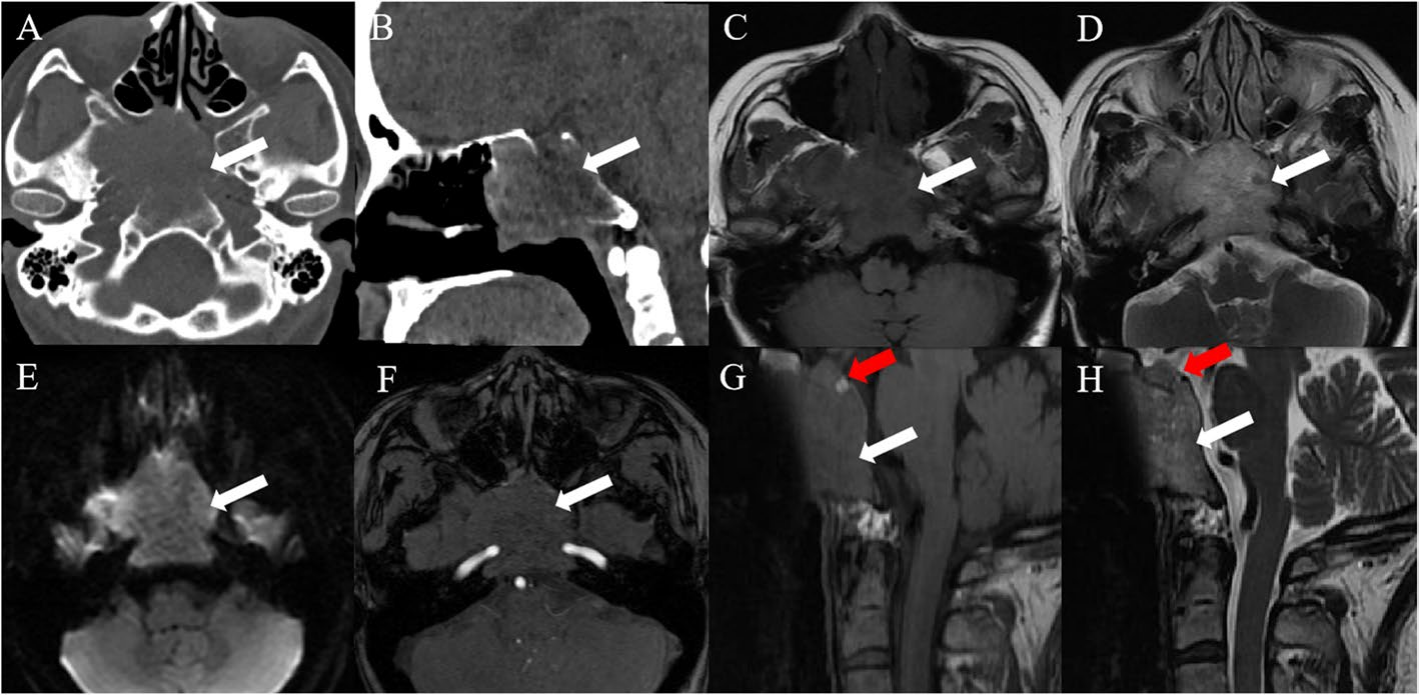
Preoperative CT and MRI. **A** Axial CT image without contrast demonstrates an ill-defined mass (white arrow) in the sphenoid sinus and clivus eroding the adjacent bone. **B** Sagittal CT image displays the relationship between the mass (white arrow) and adjacent structures. **C-F** The mass (white arrow) presents as low to high signal on T1-weighted and T2-weighted images with slightly diffusion restriction and vessels involvement. **G**-**H** The mass (white arrow) was outside the sellar without a direct connection to the normal pituitary tissue (red arrow)

**Fig. 2:**
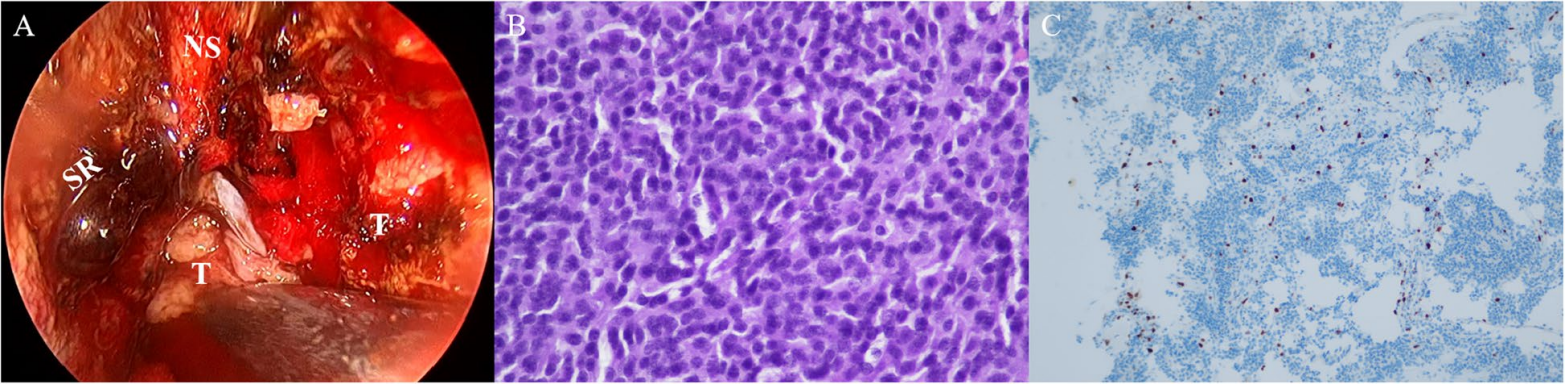
Intraoperative videocaptured photograph and pathology. **A** The patient accepts partial tumor resection via transsphenoidal endoscopy. **B** Hematoxylin-eosin staining reveals pituitary adenoma (×400). **C** The proliferation index, expressed as a percentage of Ki-67 antigen-positive nuclei, is around 3% of cells (×200). Note: SR = sphenoethmoidal recess, NS = nasal septum, T = tumor

**Fig. 3:**
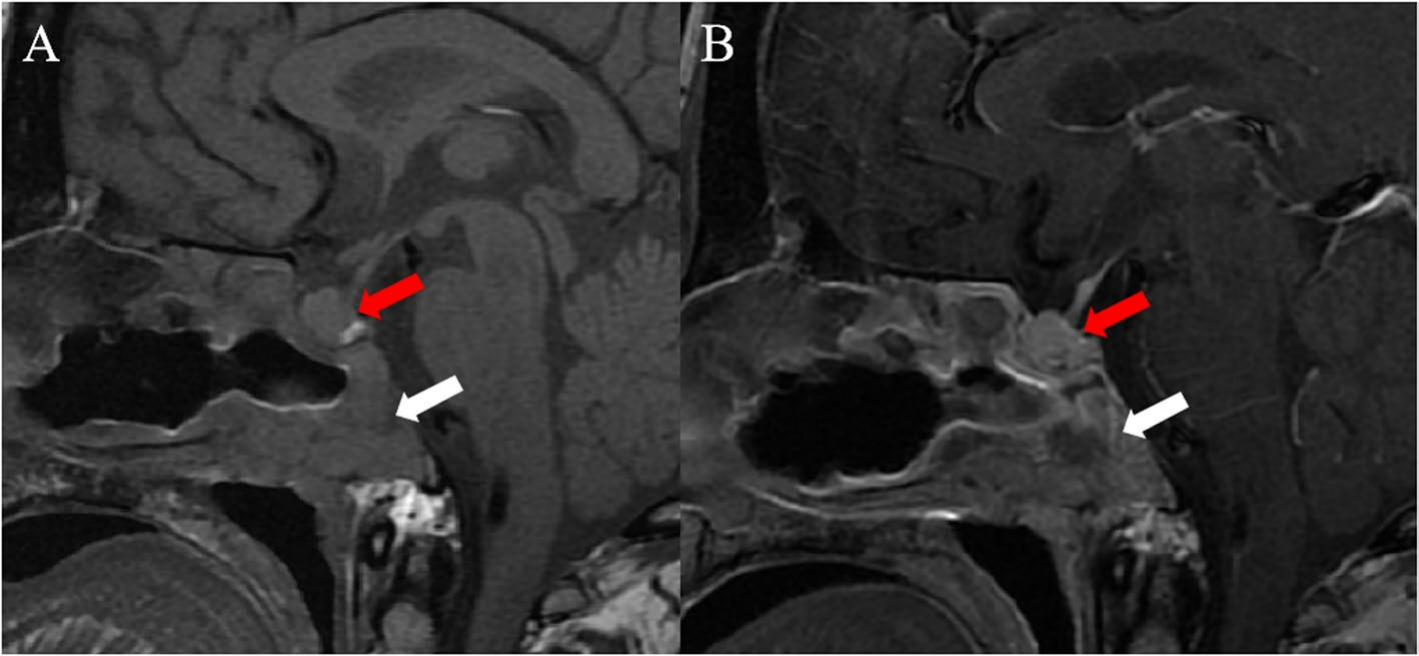
Postoperative pituitary MRI. Postoperative pre- (**A**) and post-contrast (**B**) sagittal T1-weighted images reveal the residual viable tumor (white arrow), with isointense on T1-weighted images and moderate enhancement, without connection to the normal pituitary tissue (red arrow)

A 47-year-old female was admitted to our hospital with a ten-day history of hematuria. During admittance, the patient presented with bloody nasal discharge, dizziness and headache. Paranasal sinus CT and brain MRI scans were performed. The patient denied weight gain or loss during recent years. The cardiopulmonary and neurological examinations were normal. The antithyroglobulin antibody (TGAB) level was markedly increased to 1156 IU/ml (normal range, 0-115 IU/ml). All serum tumor markers were within the normal ranges. A non-contrast CT scan of the paranasal sinus revealed an ill-defined mass (mean CT value, 38 HU) in the sphenoid sinus and clivus, eroding the adjacent bone (Fig. 1A-B). The mass did not contain any necrosis, hemorrhage or calcification. Brain MRI showed a heterogeneous mass with hypointensity on T1-weighted images, hyperintensity on T2-weighted images and isointensity on diffusion-weighted images (Fig. 1C-E). The mass was 5.8 × 3.6 × 3.7 cm in size, extending both anteriorly into the sphenoid sinus and superiorly into the bilateral cavernous sinuses (Fig. 1F). The mass was located outside of the sellar turcica, without any continuity with the intrasellar pituitary gland (Fig. 1G-H). No obvious deviation of the pituitary stalk was observed. Chordoma was suspected. The patient underwent partial tumor resection via transsphenoidal endoscopy (Fig. 2A). During the operation, it was found that the mass was grey-white and closely related to the surrounding structure. The sphenoid sinus wall, sphenoid floor and clivus were destroyed to varying degrees, and the nasal mucosa was also invaded. The tumor was partially resected (Fig. 3). Postoperative histological analyses demonstrated tumor cells arranged in sheets and nests, with prominent delicate vascularized stroma, confirmed a pituitary adenoma (Fig. 2B). Immunohistochemistry was positive for synaptophysin (Syn), chromogranin A (CgA), neuronspecific enolase (NSE), and adrenocorticotropic hormone(ACTH). The proliferation index, expressed as a percentage of Ki-67 antigen-positive nuclei, was around 3% (Fig. 2C). Ectopic invasive pituitary adenoma (ACTH-secreting EPA) was confirmed. After the operation, the patient recovered well and symptoms such as headache and dizziness were relieved. The serum TGAB level was decreased to 937 IU/ml (normal range, 0-115 IU/ml). The serum cortisol and plasma ACTH were within the normal ranges. Two months after the operation, she received external beam radiation with a total dose of 50.4 Gy in 28 fractions over 28 days.

## Discussion and conclusions

EPA occurs outside the sellar turcica, which is very rare clinically. Since it was first reported in 1909 [7], only approximately 100 such cases have been reported in the English literature [8, 9]. EPA can present both intracranially and extracranially. In most patients, EPA arises in sphenoid sinus and suprasellar regions with nonspecific symptoms [10]. EPA can also occur in other sites, such as the clivus, cavernous sinus, parasellar, nasal cavity, nasopharynx and the third ventricle [11, 12]. In the present case, a mass of 5.8 × 3.6 × 3.7 cm in size was found located in the sphenoid sinus and clivus. It destroyed bone, invaded muscle and encased blood vessels. The patient presented with bloody nasal discharge and headache. Several hypotheses regarding the origin and pathogenesis of EPA have been proposed, but the exact etiology is still controversial. It is broadly accepted that the tumor arises from the embryonic residues of pituitary cells along the path of migration of Rathke’s pouch [6, 13]. The ectopic anterior pituitary cells deposit in these sites and then undergo tumor transformation, resulting in the formation of EPA.

Similar to intraseller pituitary adenomas, EPA may also be classified as either functioning or nonfunctioning tumors. Functioning EPA is relatively easy to determine due to its hormonal symptoms. The endocrine disorder caused by EPA is directly related to its secreted hormone type, including Cushing’s syndrome, acromegaly, amenorrhea, lactation, decreased libido and hyperthyroidism. In previous reports [8, 14], the majority of EPA cases were functioning tumors. The patient in our case demonstrated ACTH immunoreactivity but with nonspecific symptoms. Although lack of full-workups of pituitary function, it is similar to a previous case report [15]. This type of tumor is usually incidentally discovered in routine imaging for other conditions. EPA may show aggressive behavior. Bone invasion [15], tumor seeding [16], and malignant transformations [17–19] have also been reported in several case series. The mechanism of this aggressive behavior is unclear. Bone invasion or erosion is common and found in most reported malignant cases. In our case report, three features (destroying bone, invading muscle, encasing blood vessel) were consistent with the aggressive criteria.

Preoperative imaging examination plays an important role in the evaluation of EPA. The typical imaging phenotype of EPA can be deciphered from a comprehensive literature review of published reports. Compared to CT, MRI has higher soft tissue resolution and is the first choice for EPA. MRI can clearly show the location, size and extent of the tumor, as well as the relationship between tumor and normal pituitary tissue, which provides important guidance for preoperative localization, treatment management and prognosis evaluation. The MRI findings of EPA are not characteristic, therefore accurate preoperative diagnosis remains a challenge. In the tumor, small foci of hypointensity on T1-weighted images and hyperintensity on T2-weighted images are reported [20]. Histologically, these foci correspond to enlarged spaces filled with secreting granules. The enhancing solid components and non-enhancing secretion filled space are mixed to form a characteristic cribriform pattern, which may be a suggestive sign. When differential diagnosis is difficult, surgical intervention and biopsy should be considered.

Treatment of EPA is similar to intrasellar pituitary adenomas and is dependent on the patient’s clinical manifestation and tumor size, location and invasion. For cases with noticeable endocrine changes or severe local compression, surgical treatment should be considered but individualized according to the specific location of the lesion. Tumor occurrence or poor surgical candidates may be supplemented by radiotherapy and drug therapy [21, 22]. In our case, the patient underwent partial tumor resection. The follow-up MRI-scan revealed residual, viable tumor and the patient accepted stereotactic radiosurgery.

Because of the rarity of this disorder, accurate preoperative diagnosis for EPA remains challenging. The final diagnosis depends on the combined application of clinical symptoms, imaging phenotype, histology and immunohistochemical markers. Treatment management should be individualized. Our case report advances our understanding of the imaging phenotype of EPA on CT and MRI.

## Data Availability

All data and materials gathered during this study are included in this study.

## Abbreviations

EPA: Ectopic pituitary adenoma
TGAB: The antithyroglobulin antibody
Syn: Synaptophysin
CgA: Chromogranin A
NSE: Neuronspecific enolase
ACTH: Adrenocorticotropic hormone.
